# Telocytes and ezrin expression in normal-appearing tissues adjacent to urothelial bladder carcinoma as predictors of invasiveness and recurrence

**DOI:** 10.1038/s41598-023-33282-0

**Published:** 2023-04-15

**Authors:** Mohamed Wishahi, Sarah Hassan, Marwa Hassan, Mohamed Badawy, Ehab Hafiz

**Affiliations:** 1 Department of Urology, Theodor Bilharz Research Institute, Kornaish El Nile, Warrak El-Hadar, Imbaba, P.O. 30, Giza, 12411 Egypt; 2 Department of Electron Microscopy, Theodor Bilharz Research Institute, Kornaish El Nile, Warrak El-Hadar, Imbaba, P.O. 30, Giza, 12411 Egypt; 3 Immunology Department, Theodor Bilharz Research Institute, Giza, Egypt

**Keywords:** Bladder, Cancer

## Abstract

Recurrence and progression rates vary widely among different histological subtypes of bladder cancer (BC). Normal-appearing mucosa in non-muscle invasive bladder cancer and muscle-invasive bladder cancer in cystoscopy and histopathology is a factor in staging and treatment. Telocytes (TCs) are spindle-shaped cells that connect with other cell types allowing communication though cytoskeletal signaling. They are involved in tissue regeneration and pathogenesis of diseases and cancer. In this study, 12 normal-appearing tissues from urinary bladder with BC, both invasive and non-invasive were evaluated in patients who had either trans-urethral resection of bladder tumor or cystectomy. In each case, cystoscopy, intraoperative inspection, and histopathology all confirmed the absence of cancerous elements. Five patients with neurogenic bladder were used as a control group. Immunohistochemistry revealed that c-Kit + cells were intensively distributed in bladder layers from BC samples, while they were seldom detected in the control group. Ultrastructural examination of reprocessed tissue showed an intense distribution of TCs and telopodes in the submucosa and between smooth muscle cells in BC. Telopodes were numerous, arborizing, and intercommunicating. Whereas TCs and telopodes were scarce in the neurogenic bladder. Also, cancerous tissue had the highest expression level of ezrin protein, and this level gradually decreased as we moved away from the tumor. Our finding of TCs number in normal-appearing tissues in conjunction with ezrin expression may compete invasiveness and possibly a trail to reduce recurrence rates.

## Introduction

Urinary bladder cancer (BC) represents 3.0% of all newly diagnosed cancer cases each year which represents 573,000 worldwide in 2020, with 440,864 males and 132,414 females, with 212,536 new deaths and it accounts for 2.1% of all cancer deaths^1^. The global age-standardized incidence rate (per 100,000 person/year) is 9.5 in men and 2.4 in women^1,2^.

More than 80% of patients with BC present with non-muscle invasive bladder cancer (NMIBC) confined to the mucosa such as noninvasive papillary carcinoma (stage pTa) and carcinoma in situ (stage pTis) or involving the submucosa; (stage pT1), with urothelial blabber carcinoma (UBC) the most frequent histological subtype. Patients with NMIBC have long-term survival and a lower risk of cancer-specific mortality in many cases compared to patients with muscle-invasive bladder cancer (MIBC) in the pT2-4 stages^3^.

The treatment modalities for BC rely on the nature of the tumor specimen, and the status of the adjacent normal-appearing mucosa as determined by cystoscopic appearances and light microscopy^4,5^. Management of NMIBC includes transurethral resection of the visible bladder tumor (TURBT), with or without intravesical instillation of bacillus Calmette-Guérin (BCG), to prevent recurrence and progression. MIBC is treated with cystectomy and neoadjuvant or adjuvant chemotherapy. However, NMIBC has a high risk of recurrence (about 25% of cases) and progression to MIBC, which is associated with a high risk of metastases and mortality^3,6^.

Diagnosis of BC is based on the TURBT findings of the lesion visible during cystoscopy examination with or without biopsy of adjacent normal-appearing mucosa^6^. Although still controversial, mapping biopsies from the normal-appearing mucosa during cystoscopy are recommended especially in cases of positive urine cytology or non-papillary tumors, or high-grade lesions, to exclude concurrent CIS which would change the postoperative therapy and follow‐up schedule^6,7^. In this study we characterized these adjacent normal-looking biopsies that were located 2–5 cm from the original tumor.

In patients with NMIBC at first diagnosis, the normal-appearing mucosa is often the site of recurrence and cancer progression during follow-up after BCG instillation. Recurrence arises in these sites with the same or higher grade. So, normal-appearing bladder tissues in MIBC and high-risk NMIBC harbor cancerous potentials^6–8^. As a result, there is a critical need for additional biomarkers that can predict the risk of recurrence and progression in patients with early-stage tumors.

Telocytes (TCs) are special stromal cells present in various organs and tissues that play an important role in cell communication via membrane-to-membrane linkage. They form a network through interconnecting long thin cytoplasmic processes called telopodes (Tps) which are 20–80 μm monofilament prolongations that emerge from a small oval cell body^9^. They express CD117 (c-Kit), CD34, and PDGF, but none of these markers are specific immunohistochemical markers. Transmission electron microscopy (TEM) is the specific method for identifying TCs. They can be subtyped into different phenotypes based on their ultrastructural and immunohistochemical properties^10,11^. They have a distinct role in tissue regeneration, regulation of immune response, as well as the regulation of cell adhesion and the cytoskeleton in tumor stromal remodeling and progression^12–15^.

Ezrin is a protein that regulates cell networks by linking the membrane proteins and the actin filaments of the cytoskeleton. It is a member of the ERM family of proteins (ezrin, radixin, and moesin). These proteins contribute to the tumorigenesis of many cancer types by affecting cell survival, migration, and invasion^16,17^.

Accordingly, this study was conducted to investigate the expression of c-Kit + TCs and characterize their morphology and distribution, as well as to evaluate the expression of ezrin in the normal-appearing bladder tissues adjacent to the UBC to explore their potential as predictors of progression and recurrence of the tumor.

## Results

**Figure 1:**
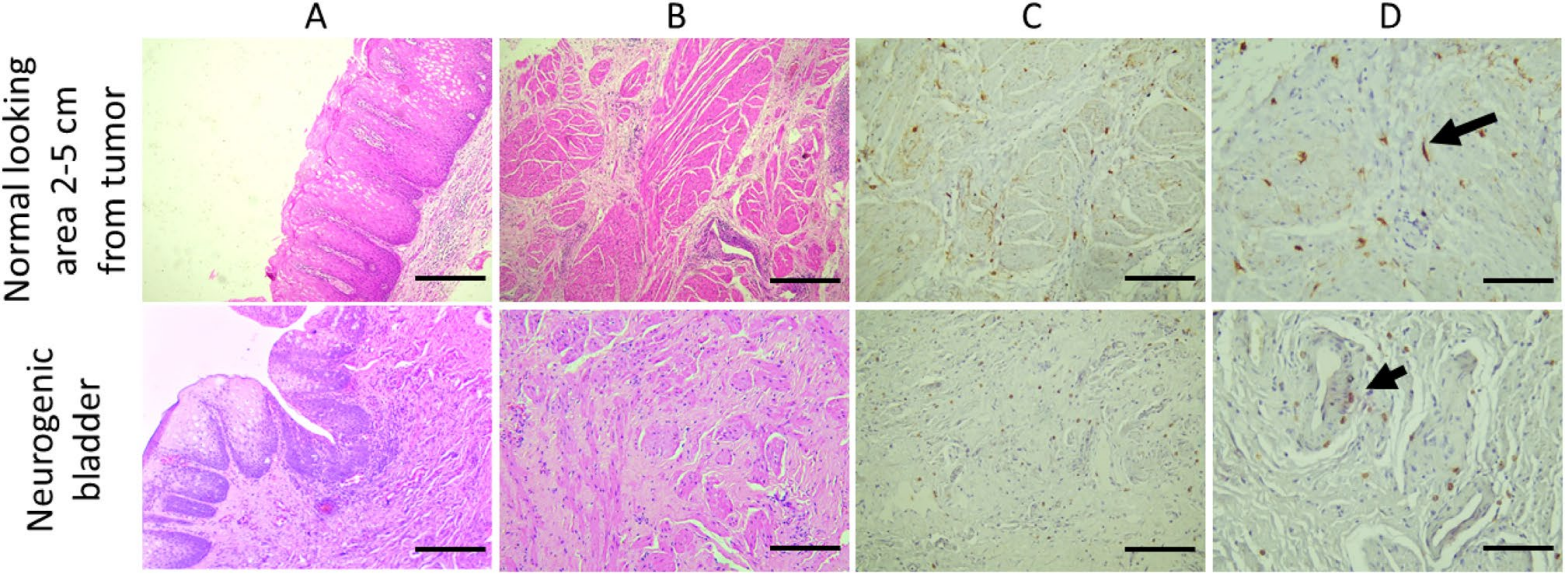
Light microscopy with H&E and cKit IHC findings in normal-appearing tissue of urinary bladder with UBC: (**A**,**B**) showed no evidence of cancer in mucosa, submucosa and muscularis propria layers. (**C**,**D**) cKit IHC shows intense distribution of cKit + ve cells in the muscle layer (long arrow). Neurogenic bladder: (**A**,**B**) showing increased collagen within the muscle layer. (**C**,**D**) cKit IHC shows of cKit + ve cells mainly close to blood vessels (short arrow). (**A**,**B**,**C**: ×100, scale bar 100 µm, **D**: 200×, scale bar 50 µm). (×200, scale bar 50 µm).

**Figure 2:**
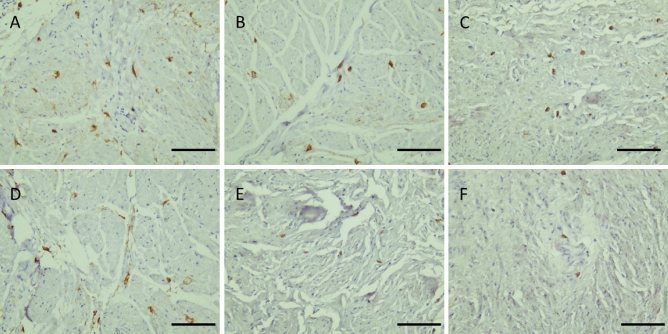
cKit IHC shows distribution of c-Kit positive cells in different samples of normal-appearing tissue of urinary bladder with UBC. (**A**,**B**,**C**): MIBC. (**D**,**E**): NMIBC, high grade and (**F**): NMIBC low grade. (× 200, scale bar 50 µm).

The normal-appearing tissues of urinary bladder with UBC revealed no evidence of cancer in the mucosa, submucosa, and muscle layer under conventional light microscopy examination with a normal distribution of collagen to smooth muscles. (Fig. 1 and Supplementary Fig. 1) Immunohistochemistry studies showed an intense distribution of c-Kit + cells in the submucosa and muscle layer (Fig. 1). This was noted in all cases of MIBC and 2 cases out of 5 with NMIBC with histological high grade (Fig. 2).

**Figure 3:**
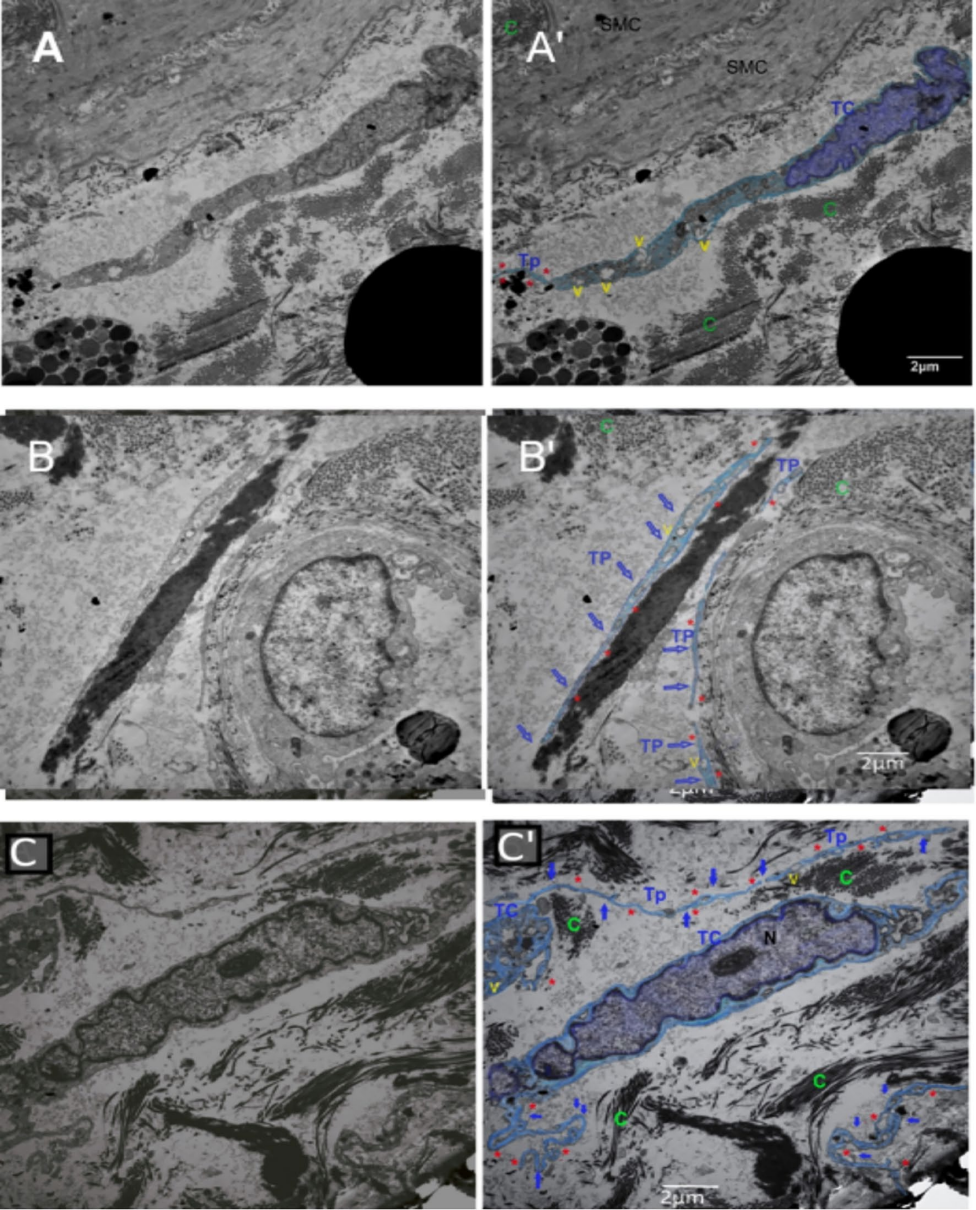
TEM microgram of submucosa and muscularis propria of normal-appearing tissue of urinary bladder with MIBC. **(A,A′)** (A: original findings), (A’: digitalized image) showing TC (TC colored light blue), TC nucleus (colored violet) with distinctive cytoplasm containing numerous vesicles (yellow V), and Tps (Tp red Asterix). TCs and Tps are infiltrating between smooth muscle cells (SMC) and dense collagen (green C). **(B,B′)** (B: original findings), (B’: digitalized image) showing Tp (colored light blue, pointed with short blue arrows, they have many vesicles (yellow V), and three Tp are in close contact with blood vessels. **(C,C′)** (**C**: original findings), (**C′**: digitalized image) showing two TC cells (colored light blue) with a distinctive nucleus in one of them (N), network of Tps appearing numerous, branching, and arborizing (small blue arrows and red Asterix), Tps cytoplasm are rich in vesicles (yellow V), TCs and Tps are infiltrating dense collection of collagens (green C). (Scale bar 2 μm).

**Figure 4:**
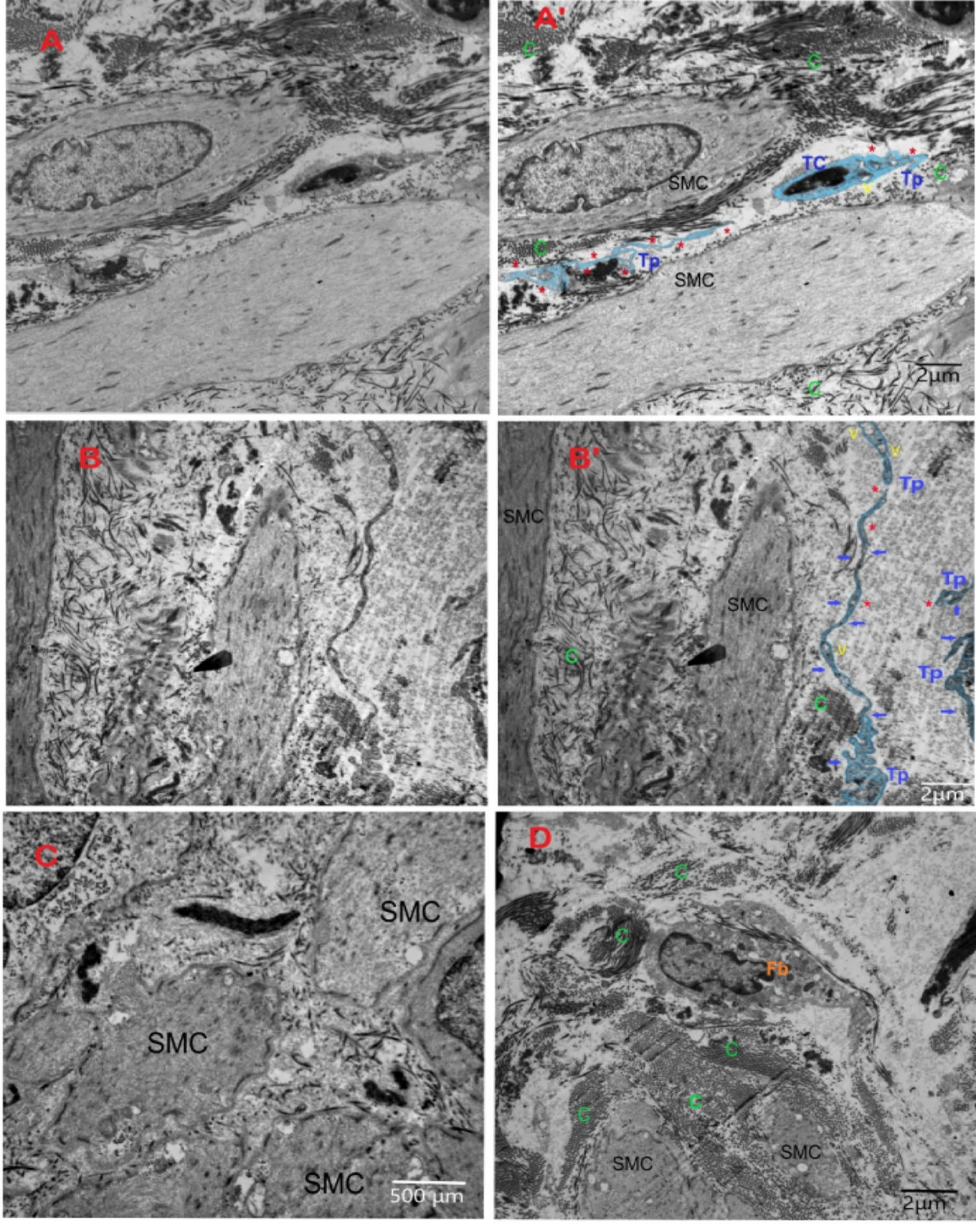
TEM microgram of submucosa and muscularis propria of normal-appearing tissue of urinary bladder with NMIBC. **(A**,**A′)** (**A**: original findings), (**A’**: digitalized image) showing TC with its distinct nucleus, and Tp (red Asterix) appearing thin, tortuous, and having numerous vesicles (V). TC and Tp are insinuated between two smooth muscle cells (SMC) and abundant collagen (green C). (Scale bar 2 μm). **(B**,**B′)** (B: original findings), (**B′**: digitalized image) showing Tp (blue short arrows and red Asterix) that has numerous vesicles (yellow V) and is attached to the smooth muscle cell (SMC) and collagen (green C). (Scale bar 2 μm). **(C**,**D)** showing smooth muscle cells (SMC) with condensation of collagen (c) between smooth muscle cells (SMC), and fibroblast cell (Fb) in close contact. No TCs or Tps were found. (Scale bar C:500 nm, D:2 μm).

Although both mast cells and TCs are c-kit positive, under TEM, each has different ultrastructural features, and it is relatively easy to confirm and differentiate between these two types of cells. Mast cells are large cells with round nuclei and abundant cytoplasm containing electron-dense cytoplasmic granules while TCs are small spindle shaped with spindle shaped nuclei and long cytoplasmic processes; Tps (Supplementary Fig. 2). Tps are intercommunicating varicose-like processes characterized by their numerous vesicles and mitochondria. The ultrastructural study detected frequent distribution of TCs with their fine arborizing Tps. They were noted both in the submucosa and muscle layers, mainly in close contact with smooth muscle cells (SMCs) and myofibroblasts with Tps extending far enough to form a network connecting SMCs over large areas (Figs. 3, 4).

**Figure 5:**
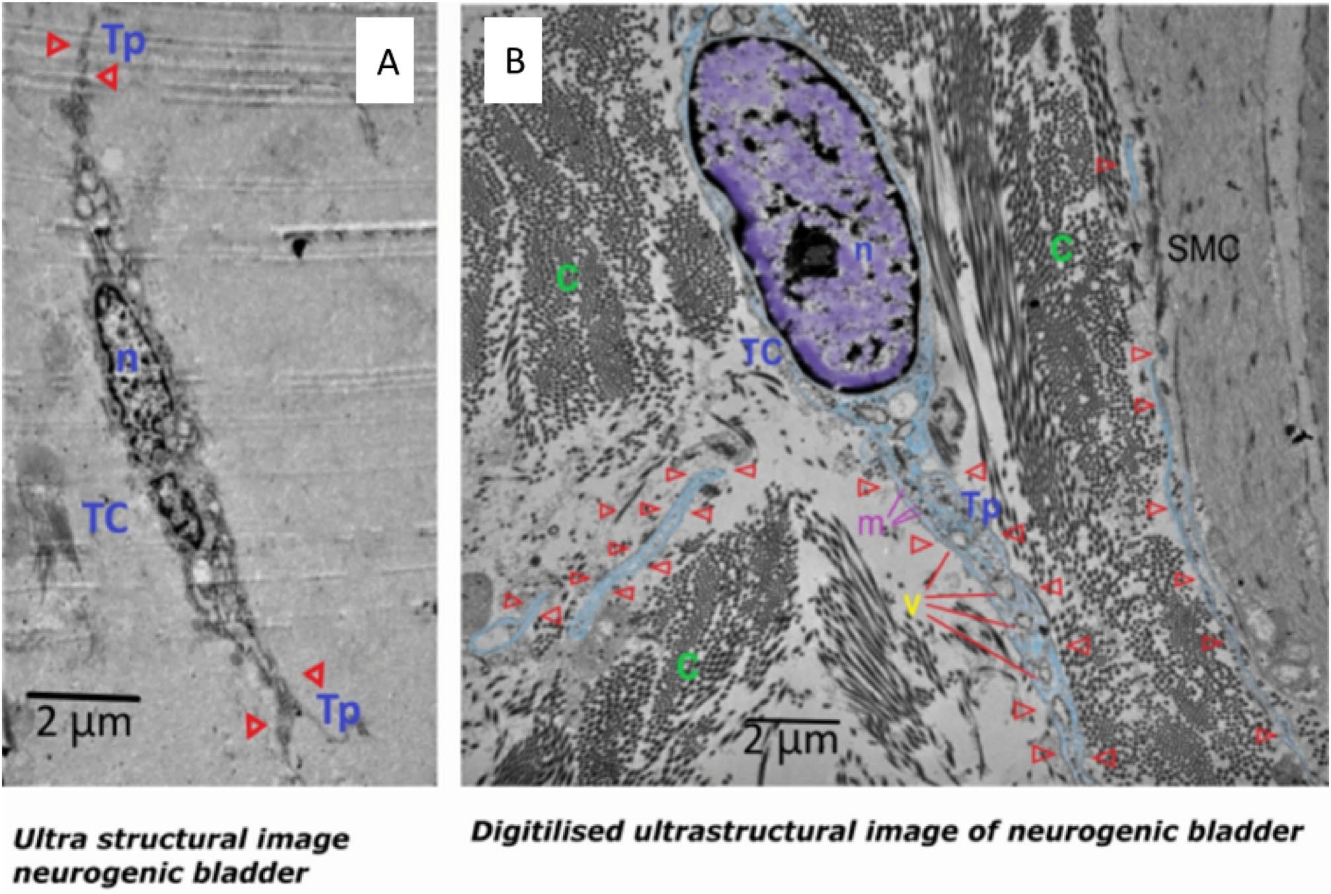
Findings in urinary bladder in cases of neurogenic bladder dysfunction. (**A**) Transmission electron microgram shows TC with its nucleus (n) and short thin cytoplasmic extension of Tp (red triangle). (**B**) Digitalized image of ultrastructural findings in neurogenic bladder dysfunction shows TC with its nucleus (n), Tp (red arrows) having many vesicles. Telopodes appeared in close contact to smooth muscle cells (SMC) and excess collagen. (Scale bar 2 μm).

On the other hand, examination of neurogenic bladder excised tissue confirmed the absence of pathologic evidence of cancer, with decreased muscle/collagen ratio. Immunohistochemistry analysis revealed that c-Kit + cells were mainly located in the submucosa and around the blood vessels. TCs were seldomly detected in TEM examination of the neurogenic bladder tissues (Fig. 5).

**Figure 6:**
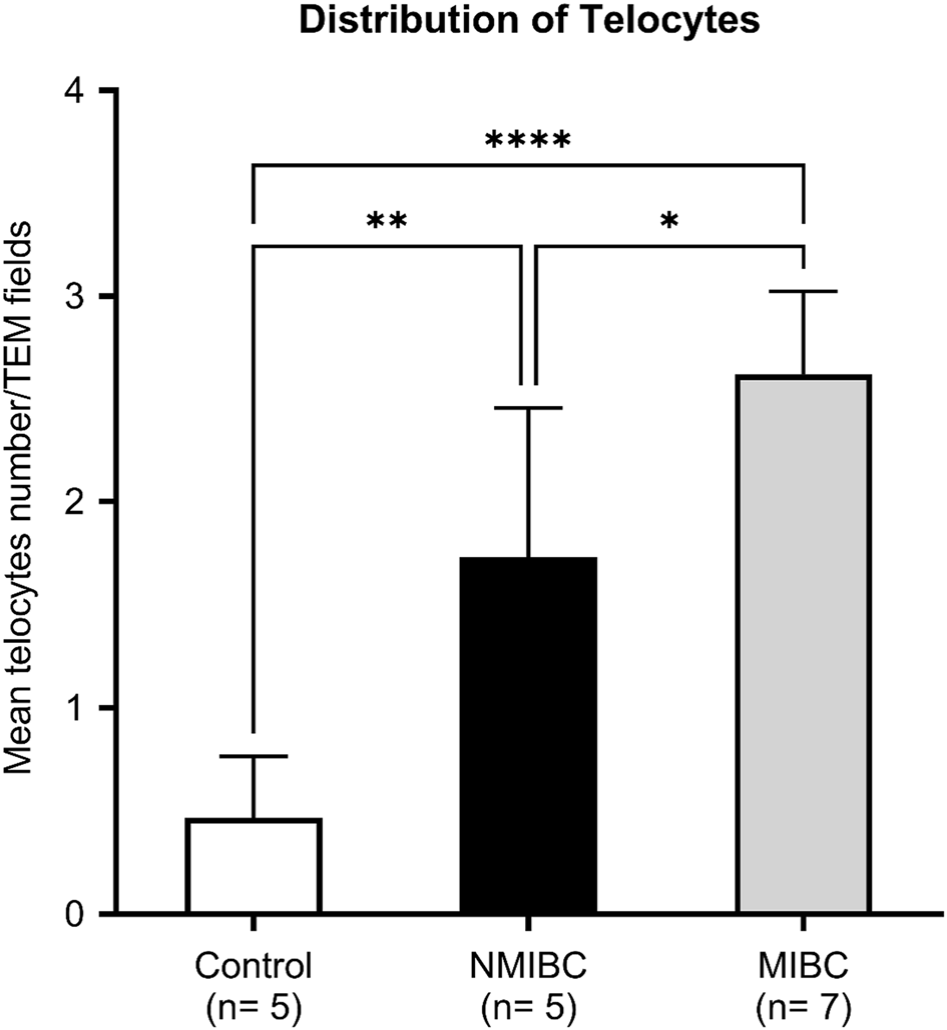
Relative quantitative distribution of TCs/TEM fields. Results are expressed as mean ± SD, ****p < 0.001, **p < 0.01, *p < 0.05.

Distribution of TCs and configuration of Tps was detected in all samples of normal-appearing tissues in cases of MIBC with a mean of 2.6 ± 0.4 cells/TEM fields and a mean of 1.7 ± 0.7 cells/TEM fields of NMIB compared to 0.4 ± 0.2 cells/TEM fields of the control group (*p* < 0.001 and *p* < 0.01, respectively) (Fig. 6).

**Figure 7:**
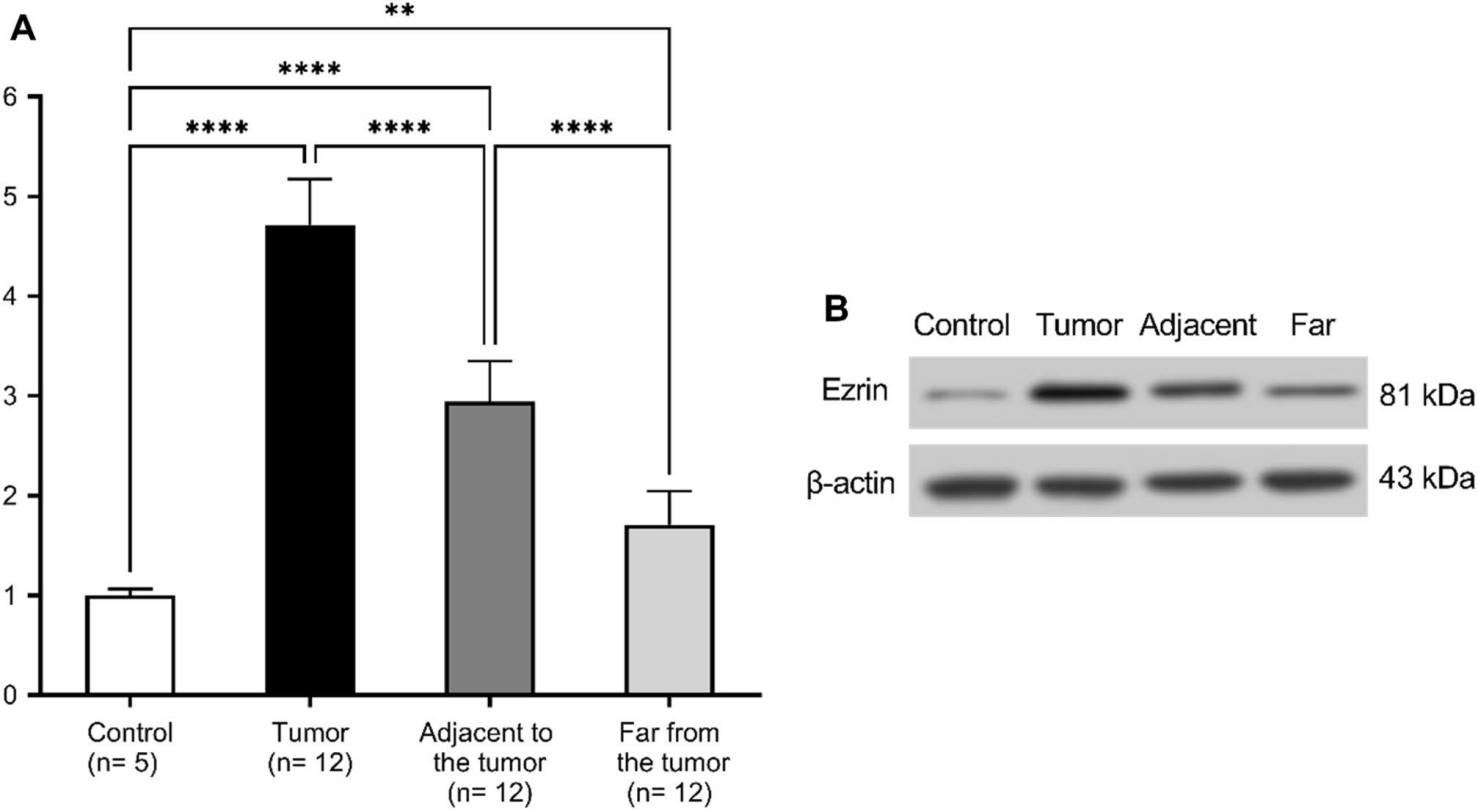
(**A**) Relative expression levels of ezrin protein. (**B**) Western blot images for ezrin expression. Results are expressed as mean ± SD, ****p < 0.001, **p < 0.01.

Also, the western blot examination revealed that the cancer tissue had the highest expression level of ezrin protein, and this level gradually decreased as we moved far from the tumor. However, its expression levels in tissues distant from the tumor site remained significantly higher than in the control group (*p* < 0.01) (Fig. 7) (original blot is presented in Supplementary Fig. 3).

## Discussion

The strategic plan for treating NMIBC has been considered the standard treatment and was successful in the majority of cases. However, a proportion of cases experienced recurrence, progression, and metastases. This group of patients appeared to have normal mucosa during cystoscopy^6,7^. Several reports have revealed that the normal-appearing mucosa in BC was not normal and may had concurrent dysplasia or CIS when examined with light microscopy^7,8,21,22^. Hence, there is a great need for additional tools and biomarkers to examine the normal-looking tissues in NMIBC to assess the likelihood of progression and recurrence, as well as, in MIBC patients who require proper staging for initiation of chemotherapy, radiotherapy, or immunotherapy.

The expression of TCs in BC is still an unresolved issue. Our analysis shows that TCs were not equally expressed in all cases of BC denoting a different neoplastic pattern. In the present study, the normal-looking mucosa in the urinary bladder with UBC was reconsidered and evaluated by examining the normal-appearing tissues with IHC and EM studies. We found that the c-Kit + TCs were intensively expressed in MIBC, but not in all cases of NMIBC.

Nimphius et al. and others have misinterpreted strong c-Kit signals from the tumor-free stroma as mast cells, which were found scattered throughout the tumor-free lamina propria and tunica muscularis. This was due to the lack of ultrastructure studies^11,23^. Indeed, both mast cells and TCs are c-Kit positive. Proper interpretation requires awareness of their distribution and morphology. Although both can share same distribution, mast cells are usually located in the submucosa and around the blood vessels. While our cells of interest are the interstitial cells, located mainly in contact with myocytes within the muscularis propria^24^. In order to make a precise interpretation, we reprocessed the tissue from the paraffin blocks using a special technique for electron microscopy examination^25^. A selection mechanism similar to that used for tissue microarray, in which the histological and IHC examinations are performed first, followed by the selection of the area of interest to be examined under TEM where these two types of cells can be easily distinguished. (Supplementary Fig. 2).

Growing evidence suggests that ezrin is an oncogenic protein because it is associated with invasive and metastatic behaviors in many types of malignancies. It mediates signal transductions in tumorigenesis and diverse cellular processes via interactions with a variety of adhesion molecules and growth factor receptors, as well as it acts through cytoskeletal reorganization^16^. Several studies, though still controversial, have shown that positive ezrin expression is a predictor of a good prognosis in BC in terms of overall survival^16–20^. Our findings revealed that the highest level of ezrin protein expression was found in cancer tissue and that this level gradually decreased as we moved away from the tumor indicating its potential role in the development or progression of BC.

A multifocal growth pattern is frequently seen in bladder cancer^26^. The findings of the current study indicate that the normal-looking tissue adjacent to the BC is not normal. This is confirmed by previous reports that detected cancerous lesions ranging from CIS, dysplasia, and positive tumor in random biopsies taken from normal-appearing mucosa in the bladder with either MIBC or NMIBC. This could be attributed to a disruption of the physico-chemical microenvironment of the tissues which leads to cell differentiation and phenotype transformation toward cancer^27^. The assumed pro-proliferative effects of TCs would most likely be due to their activation in response to the physico-chemical changes in the microenvironment^28^.

To the best of our knowledge, this is the first study to investigate the density and distribution of c-Kit + TCs in the normal-appearing tissue of the urinary bladder with UBC using immunohistochemistry and reprocessed TEM for ultrastructural studies. The controversial role of TCs between regeneration and supportive roles in organ and tissue homeostasis on one side and promoting cancer proliferation on the other warrants considering TCs as either a guard against tumor progression and recurrence or a contributor to tumorigenesis. Our findings regarding the frequency of TC expression coupled with ezrin may help to reduce invasiveness and recurrence rates.

## Methods

### Participants’ collection

Twelve patients were included in this study, 9 males and 3 females. They underwent radical cystectomy for MIBC stage pT2-3 (n = 7), and transurethral resection (TURBT) for NMIBC pT1 (n = 5). Only cases with urothelial type of bladder carcinoma either with or without muscle invasion (MIBC, NMIBC) are included and other forms of tumors or malignancy were excluded. They did not receive neoadjuvant chemotherapy and had BC as a single primary tumor with no metastasis. Their age range was 42–72 years, with a mean age of 54.5 ± 8.8 years. The surgical specimens were representing whole bladder wall layers in areas where the mucosa appeared normal, including subepithelial tissue and part of the superficial muscle layer. These samples are taken at a distance, at least 2–5 cm away from the tumor margins to avoid the presence of dysplasia or CIS, desmoplastic reaction or tumor extension into the deeper layers. Five surgical specimens from urinary bladder with neurogenic bladder dysfunction (NBD), underwent constructive surgery with excision a segment of the dome of the bladder wall, were used as controls for non-malignant bladder.

The institutional review board (IRB) of Theodor Bilharz Research Institute approved the present study (Research Ethics Committee: (TBRI- Protocol No: PT 722). This study was conducted in compliance with the relevant laws and regulations, as well as good clinical practice and ethical principles described in the World Medical Association’s Declaration of Helsinki. The requirement for patients to provide informed consent was waived by the IRB of Theodor Bilharz Research Institute because of the retrospective nature of the study.

### Histological examination

The 17 surgical specimens were processed for histological examination with Hematoxylin and Eosin (H&E) and Masson trichrome (MTC) stains. H&E examination was done to detect the cancer changes and to map cancer margins and free areas.

### Immunohistochemistry analysis

Immunohistochemistry (IHC) was performed on 5 µ-thick sections of formalin-fixed and paraffin-embedded tissue samples. Sections on charged slides are treated with Rabbit polyclonal anti-CD117 (c-kit) antibodies (CD 117, Cat. No. A4502, Dako, Denmark) as the primary antibody to label the c-kit positive cells and evaluate their density and configuration. Goat anti-mouse biotinylated immunoglobulins/HRP (Cat. No. P0447, Dako, Denmark) were utilized as the secondary antibody. Streptavidin–biotin–peroxidase complex and peroxidase-DAB (3,3′-diaminobenzidine) (Dako, Denmark) were applied. All were performed according to the manufacturer’s instructions. Positive and negative control slides were included in each run.

### Transmission electron microscopic examination

Transmission electron microscopy (TEM) studies were performed using a retrospective technique similar to that used for tissue microarray to select specific areas of formalin-fixed, paraffin-embedded blocks^25^. After matching the histological and IHC tissue sections to the paraffin block, extraction of cylindrical tissue cores containing the area of interest was done from the paraffin block. The areas of interest were de-waxed in three xylene washes before being re-hydrated in descending-graded ethanol. The selected samples were refixed in a glutaraldehyde and cacodylate buffer mixture, washed in cacodylate-sucrose buffer, and post-fixed in osmium tetroxide at 4 °C in a cold room. The samples were dehydrated in ascending graded ethanol prior to being impregnated in Epon 812 substitute (EMBed-812 Kit, Electron Microscopy Science, USA) at room temperature and polymerized at 60 °C for 48 h. Semithin sections were cut first from the resin block to confirm the presence of tissue in request. Then ultrathin sections were made using an Ultracut R ultramicrotome (Leica, Vienna, Austria). The mounted grids were examined at 80 kV under Jeol TEM Jem-1400 electron microscope (Jeol, Japan). We categorized each case according to TCs and/or Tps count detected in 3 examined TEM fields into: (0) none, (1) at least one was detected. (2) 2 and < 4 (3) more than 4.

### Western blot examination

The surgical specimens from each group were homogenized in phosphate-buffered saline (PBS). The total proteins were extracted from the homogenized tissues using the ReadyPrepTM protein extraction kit provided by Bio-Rad Inc (Catalog #163–2086) according to the manufacturer’s instructions. To determine the protein concentration in each sample, the Bradford Protein Assay Kit (SK3041) was utilized. Then, 20 μg protein from each sample was mixed with an equal volume of 2 × Laemmli sample buffer which contained 4% SDS, 10% 2-mercaptoehtanol, 0.004% bromophenol blue, 20% glycerol, and 0.125 M Tris HCl. Each mixture was boiled at 95 °C for 5 min to ensure protein denaturation. The samples were then run through polyacrylamide gel electrophoresis (PAGE) before being transferred to a polyvinylidene difluoride (PVDF) membrane. The membrane was blocked with tris-buffered (TBST) saline in Tween 20 and 3% bovine serum albumin (BSA) for 1 h. Incubation was done overnight in the primary antibody against ezrin protein at 4 °C. The blot was rinsed with TBST 3–5 times for 5 min. The blotted target protein was incubated in the HRP-conjugated secondary antibody solution (Goat anti-rabbit IgG- HRP) for 1 h. The blot was rinsed with TBST 3–5 times for 5 min.

The chemiluminescent substrate (Clarity TM Western ECL substrate Bio-Rad cat#170–5060) was applied to the blot where equal volumes were added from solution A (Clarity western luminal/enhancer solution) and solution B (peroxidase solution). A CCD camera-based imager was used to capture the chemiluminescent signals. Image analysis software was used to read the band intensity of the target proteins against the control sample β-actin (housekeeping protein) on the ChemiDoc MP imager.

### Statistical analysis

GraphPad Prism (version 8.0.0, USA) was used to analyze the data. All results were presented as mean ± standard deviation (SD). One-way analysis of variance (ANOVA) was used, followed by the Tukey multiple-comparison post hoc test to compare between the different groups. A *p* value < 0.05 was considered statistically significant.

## Supplementary Information

Supplementary Information.

## Data Availability

Data presented and analyzed in this study are included in this published article.
