# Supplementary material for: Telocytes and ezrin expression in normal-appearing tissues adjacent to urothelial bladder carcinoma as predictors of invasiveness and recurrence
Source: Sci Rep. 2023 Apr 15;13:6179. doi: 10.1038/s41598-023-33282-0 (PMC10105776; doi:10.1038/s41598-023-33282-0)
Supplement: Supplementary file 1 — Supplementary Information. [file 41598_2023_33282_MOESM1_ESM.docx]

Supplementary Figure 1


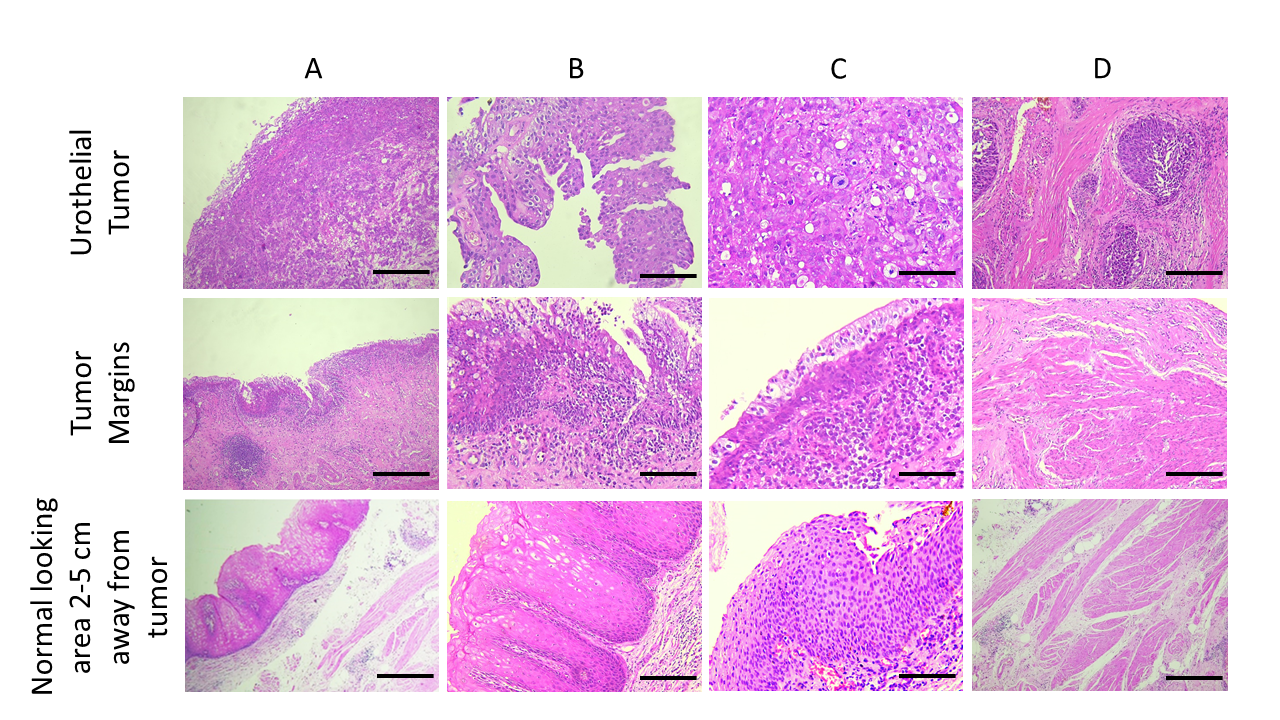


**Supplementary Figure 1**: Pictures from tumor, tumor margins and normal looking tissue 2-5 cm away from UBC to illustrate our target tissue for sampling. In the tumor area: the mucosa showed (A) non-papillary and (B) papillary types of UBC, (C) showed high grade features with abnormal mitotic figures and (D) showed invasive tumor into the muscle layer. The tumor margins showed mucosa with focal ulceration and inflammation (A-C) with muscle layer free of tumor (D). The normal looking area showed free mucosa (A-C) and muscle layer (D). (x100, scale bar 100 µm)

Supplementary Figure 2


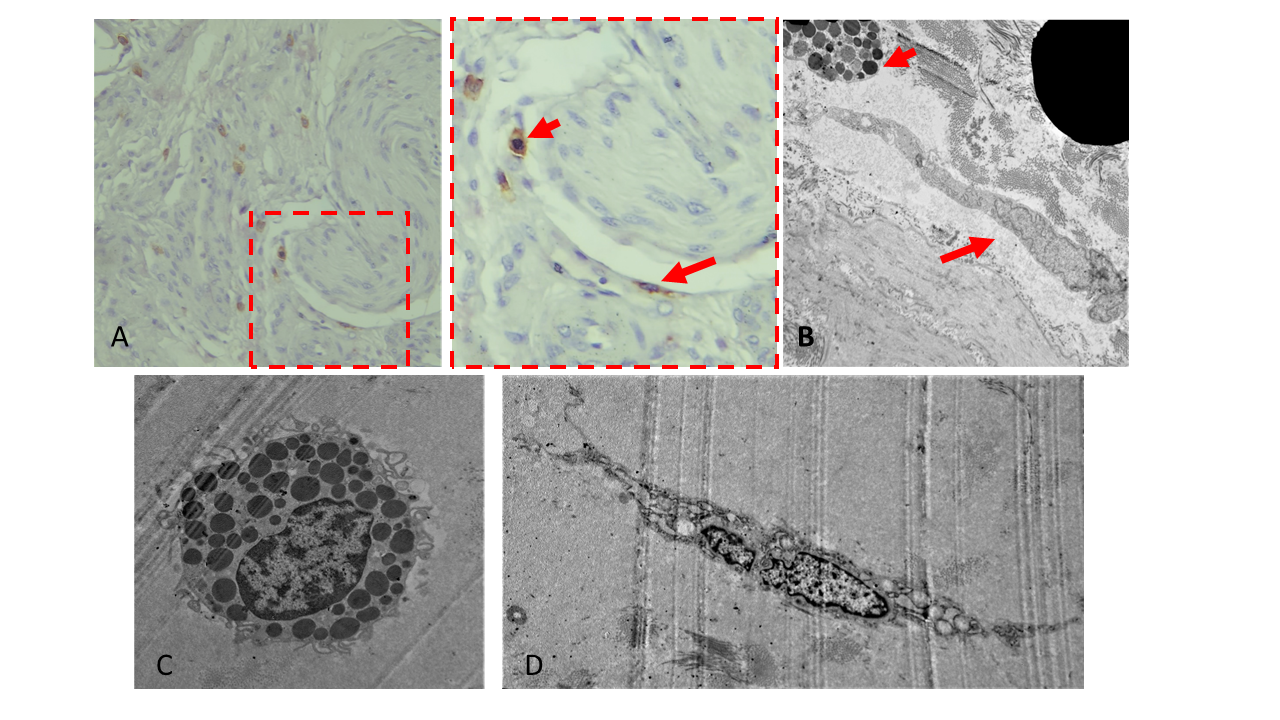


**Supplementary Figure 2**: Pictures best illustrate the morphologic difference between TCs and mast cells. (A) After matching the histological and IHC tissue sections to the paraffin block. An expert eye can appreciate the difference between TCs (long arrow) and mast cells (short arrow) even in IHC sections (Inset). Extraction of cylindrical tissue cores containing the areas of interest is done from the paraffin block and re-embedded in resin block for TEM (B). Each cell has different ultrastructural features. Mast cells are large cells with round nuclei and abundant cytoplasm containing electron-dense cytoplasmic granules (C) while TCs are small spindle shaped with spindle shaped nuclei and long cytoplasmic processes (D). (Pictures were cropped for illustration)

Supplementary Figure 3


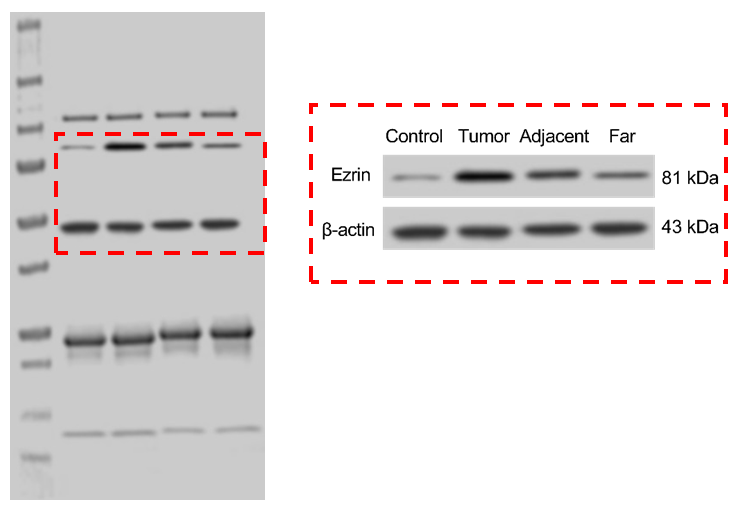


Supplementary Figure 3: Original Western Blot images for ezrin expression.
